# A Multi-Gene Model Effectively Predicts the Overall Prognosis of Stomach Adenocarcinomas With Large Genetic Heterogeneity Using Somatic Mutation Features

**DOI:** 10.3389/fgene.2020.00940

**Published:** 2020-08-26

**Authors:** Xianming Liu, Xinjie Hui, Huayu Kang, Qiongfang Fang, Aiyue Chen, Yueming Hu, Desheng Lu, Xianxiong Chen, Yejun Wang

**Affiliations:** ^1^Department of Gastrointestinal Surgery, Shenzhen People’s Hospital, The Second Clinical Medical College of Jinan University, Shenzhen, China; ^2^School of Basic Medicine, Shenzhen University Health Science Center, Shenzhen, China

**Keywords:** stomach adenocarcinoma, prognosis, prediction, multi-gene model, heterogeneity

## Abstract

**Background:**

Stomach adenocarcinoma (STAD) is one of the most common malignancies worldwide with poor prognosis. It remains unclear whether the prognosis is associated with somatic gene mutations.

**Methods:**

In this research, we collected two independent STAD cohorts with both genetic profiling and clinical follow-up data, systematically investigated the association between the prognosis and somatic mutations, and analyzed the influence of heterogeneity on the prognosis-genetics association.

**Results:**

Typical association was identified between somatic mutations and overall prognosis for individual cohorts. In The Cancer Genome Atlas (TCGA) cohort, a list of 24 genes was also identified that tended to mutate within cases of the poorest prognosis. The association showed apparent heterogeneity between different cohorts, although common signatures could be identified. A machine-learning model was trained with 20 common genes that showed a similar mutation rate difference between prognostic groups in the two cohorts, and it classified the cases in each cohort into two groups with significantly different prognosis. The model outperformed both single-gene models and TNM-based staging system significantly.

**Conclusion:**

The study made a systematic analysis on the association between STAD prognosis and somatic mutations, identified signature genes that showed mutation preference in different prognostic groups, and developed an effective multi-gene model that can effectively predict the overall prognosis of STAD in different cohorts.

## Introduction

Stomach adenocarcinoma (STAD) represents the global fifth most common malignancy and the third leading cause of cancer mortality, with estimated 1,033,701 newly diagnosed cases and 782,685 deaths in 2018 (Bray et al., 2018). Screening of STADs at early stages with endoscopy and biopsy sampling remains the most effective approach to improve prognosis and reduce the mortality (Banks et al., 2019). However, the majority of STADs worldwide except Japan and Korea were diagnosed at a late stage, due to the lack of symptoms at early stages, invasiveness of endoscopy, and unsound early-screening programs (Banks et al., 2019). Surgical resection and chemotherapy remain the main treatment regimens (Charalampakis et al., 2018). Although new therapies, such as targeted and immune therapies, have been applied to STADs, the overall outcome was only improved moderately (Tran et al., 2017; Charalampakis et al., 2018).

Multi-omics studies disclosed a high heterogeneity of STADs in genetics (Cancer Genome Atlas Research Network, 2014; Cristescu et al., 2015; Oh et al., 2018), gene expression (Boussioutas et al., 2003; Tan et al., 2011; Lei et al., 2013), and other molecular levels (Ooi et al., 2016; Ni et al., 2019; Zhang et al., 2019). The molecular heterogeneity could be associated with the complexity of anatomic regions of stomach, cell origins, and etiologies (Cancer Genome Atlas Research Network, 2014; Choi et al., 2014; Cristescu et al., 2015; Waldum and Fossmark, 2018; Ni et al., 2019; Zhang et al., 2019). STADs could originate from different anatomic sites such as cardia or gastroesophageal junction, fundus, lesser curvature, greater curvature, angular incisures, antrum, and pylorus, each with different cell compositions (Soybel, 2005; Choi et al., 2014). STADs are divided by the Lauren classification system into intestinal and diffuse types, the latter of which show poor clinical outcomes generally (Laurén, 1965; Shen et al., 2013). The World Health Organization proposed an alternative system, dividing STADs into papillary, tubular, mucinous (colloid), and poorly cohesive carcinomas (Bosman et al., 2010). Recently, genome-based molecular signatures were comprehensively identified and employed by The Cancer Genome Atlas (TCGA) to classify STADs into four subtypes, namely, Epstein–Barr virus positive (EBV), microsatellite instable (MSI), genome stable (GS), and chromosomal instability (CIN) (Cancer Genome Atlas Research Network, 2014). A gene expression-based study from the Asian Cancer Research Group (ACRG) also classified STADs into two major subtypes, MSI and microsatellite stable (MSS), while MSS STADs were further subdivided into three subtypes, epithelial-to-mesenchymal transition (EMT), TP53 active (TP53+), and TP53 inactive (TP53-) (Cristescu et al., 2015). The new molecular classification schemes could have more prospective clinical utilities in guiding STAD therapies and prognosis.

For a variety of tumors, prognosis has been reported to be associated with somatic gene mutations (Loi et al., 2013; Lee et al., 2017; Zhang et al., 2017; Cho et al., 2018; Yu et al., 2019). Despite the large heterogeneity of STADs, common genetic factors (e.g., BRCA2 and MUC16) were still identified and reported to be associated with the prognosis (Chen et al., 2015; Li et al., 2018). Currently, there is still a lack of systemic exploration of the association between STAD prognosis and somatic mutations. To achieve this goal, here, we collected the publically available data from two STAD cohorts that contained both genetic mutation profiles and clinical follow-up information (Cancer Genome Atlas Research Network, 2014; Chen et al., 2015), analyzed the STAD prognosis–genetics association globally and the influence of heterogeneity on the prognosis–genetics association, and identified a list of common genetic signatures that can be used widely for the guidance of STAD prognosis.

## Materials and Methods

### Datasets, Stratification, and Mutation Frequency Comparison

Two STAD cohorts were used in this study, the TCGA cohort and a Chinese cohort (Cancer Genome Atlas Research Network, 2014; Chen et al., 2015). The TCGA cases were multiethnic but mostly white people, while the Chinese cohort was comprised by Chinese patients exclusively. Both the clinical data and the somatic mutation data were downloaded. Mutations causing codon changes, frame-shifts, and premature translational terminations were retrieved for further analysis. For prognosis–genetics association analysis, first, the cases were removed that received targeting therapies. Furthermore, only the ones with both somatic mutation data and corresponding prognostic follow-up information were recruited. The included cases were classified into two categories according to prognosis (“good” or “poor”). The “good” prognosis group included the patients surviving through the preset follow-up period while the “poor” one indicated the patients died within the observed period. The TNM (tumor-nodal-metastasis) staging system was used for stratification, and for the sake of convenience in binary classification, two categories, “early” (Stages I and II) and “later” (Stages III and IV) were predefined. In addition, considering the possible effects of different anatomic sites of tumor on prognosis, subdivisions were used for stratification as well. To compare the somatic gene mutation frequency between prognostic groups, a matrix was prepared to record the mutations of all the genes for each case, followed by counting the number of cases with mutations for each gene in each group. A genome-wide rate comparison test (EBT) proposed recently that could balance statistic power and precision was adopted to compare the gene mutation rates (Hui et al., 2017). To test the robustness of gene mutation signatures identified by EBT tests, a repeated resampling strategy was adopted, by which a subset (70% of the total sample size) of the training cases was randomly selected for 100 rounds, gene mutation rates were compared for each round, and the signature genes were observed for the recurrence among the top 50 genes with smallest *p*-values for each round (Hui et al., 2017).

### Feature Extraction, Representation, and Model Training

Two strategies were adopted for the feature extraction in this research, *p*-value based and empirical. For the *p*-value-based strategy, the top *n* genes with the most significant mutation frequency difference were used as the genetic features. For the empirical strategy, the difference of mutation rates was calculated per gene between the two prognostic groups and ordered, and the genes with a minimal 10% (or any indicated percentage) mutation rate difference and with recurrent mutations in either group were retrieved as candidate features.

For each case, *P_*j*_ (j* = 1, 2,., *m_*i*_)* belonging to a certain category *C*_*i*_, where i equaled to 1 or 0, and *m*_*i*_ represented the total number of cases of the category *C*_*i*_, the genetic features were represented as a binary vector *F*_*j*_ (*g_1_, g_2_,., g_*n*_*) in which *g*_*k*_ (*k* = 1, 2,., *n*) represented the *k*th genetic feature, taking the value of 1 if the corresponding gene was mutated and 0 otherwise. There was an *m_*i*_^∗^n* matrix for category *C*_*i*_. When stage was used as an additional feature, the size of the matrix was enlarged to *m*_*i*_^∗^(*n* + 1), and the stage feature was also represented in a binary form in the additional column, for which 1 and 0 represented “early” and “later,” respectively. The anatomic sites were represented as two-bit features, i.e., “cardia/gastro-esophagus junction,” “fundus/corpus,” and “antrum/pylorus” being represented as “00,” “01,” and “10,” respectively.

An R package, “e1071,” was used for training SVM models using each training dataset^1^. During the training stage, all four kernels, “Radial Base Function (RBF),” “linear,” “polynomial,” and “sigmoid,” were tested and the parameters were optimized based on a 10-fold cross-validation grid search. The best kernel with optimized parameters was selected for further model training.

### Model Performance Assessment

A 5-fold cross-validation strategy was used in this study. The original feature-represented matrix for each category was randomly split into five parts with identical size. Every four parts of each category were combined and served as a training dataset while the rest one of each category was used for testing and performance evaluation.

The Receiver Operating Characteristic (ROC) curve, the area under the ROC curve (AUC), Accuracy, Sensitivity, Specificity, and Mathews Correlation Coefficient (MCC) were utilized to assess the predictive performance. In the following formula, Accuracy denotes the percentage of both positive instances (“good prognosis”) and negative instances (“poor prognosis”) correctly predicted. Specificity and Sensitivity represent the true negative rate and true positive rate, respectively, while the default threshold value from “e1070” (0.0) was used to define the Sensitivity and Specificity in the research. An ROC curve is a plot of Sensitivity versus (1 – Specificity) and is generated by shifting the decision threshold. AUC gives a measure of classifier performance. MCC takes into account true and false positives and false negatives and is generally regarded as a balanced measure which can be used even if the classes are of very different sizes.

Accuracy=(TP+TN)/(TP+FP+TN+FN),

Specificity=TN/(TN+FP),Sensitivity=TP/(TP+FN),

$$\mathrm{MCC}=\left(\left({\mathrm{TP}}^{*}\mathrm{TN}\right)-\left({\mathrm{FN}}^{*}\mathrm{FP}\right)\right)/\mathrm{Sqrt}({\left(\mathrm{TP}+\mathrm{FN}\right)}^{*}$$

$${\left(\mathrm{TN}+\mathrm{FP}\right)}^{*}{\left(\mathrm{TP}+\mathrm{FP}\right)}^{*}\left(\mathrm{TN}+\mathrm{FN}\right)).$$

### Survival Analysis

The follow-up survival information of STAD cases was annotated. To evaluate the survival of prediction results of each model, all the 5-fold cross-validation testing results were collected and grouped, followed by the survival analysis for each predicted group. Kaplan–Meier overall survival analysis was performed with R survival package^1^. The Gehan–Breslow–Wilcoxon test was used to compare the difference of overall survival curves, and the significance level was set as 0.05.

### TML Analysis

Both Tumor Mutation Load (TML) and Missense TML were analyzed for STAD cases of different prognostic groups. TML is defined as logarithm transformation of mutation rate per megabase, while Missense TML only counts the mutations causing amino acid changes. The Wilcoxon rank-sum test was performed to compare the distributions of TML or Missense TML, with the preset significance level as 0.05.

## Results

### Somatic Mutation Profile Difference Between Prognostic Groups of TCGA STADs

In total, 142 TCGA STAD cases remained after filtering the duplicates, the ones missing somatic mutation or clinical information and those treated with targeting therapies. The general clinical properties are shown in Supplementary Table S1. A somatic mutation profile analysis for these cases disclosed a list of genes with high mutation rates (>30%), including *TTN*, *PCDHAC2*, *PCDHGC5*, *TP53*, *MUC16*, *SYNE1*, and *CSMD* (Supplementary Table S2). The cases were also stratified according to sex and anatomic site, and the somatic mutation profiles were compared among the corresponding strata. Six genes were found with significant somatic mutation rates between male and female (EBT, *p* < 0.05), while 5 genes showed marginally significant somatic mutation rates among different anatomic sites (EBT, *p* < 0.10) (Supplementary Table S2).

**FIGURE 1:**
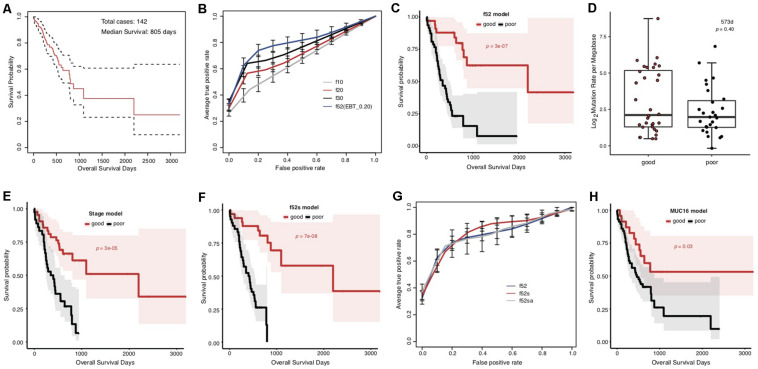
Association between the overall prognosis of TCGA STADs and somatic gene mutations. **(A)** The Kaplan–Meier overall survival curve of the TCGA STAD cases. The median was indicated with an arrow. The analysis throughout the figure used the median survival as stratification cutoff of STAD prognosis. **(B)** The 5-fold cross-validated ROC curves of genetic models predicting the prognosis of STADs. **(C)** The Kaplan–Meier overall survival curves of the TCGA STAD cases classified by f52 with a 5-fold cross-validation strategy. **(D)** The distribution of TMLs for TCGA cases of good and poor prognosis groups. The *p*-value of the Wilcoxon rank-sum test was indicated. **(E,F)** The Kaplan–Meier overall survival curves of the TCGA STAD cases stratified by TNM stage information **(E)** or the combined f52s model with a 5-fold cross-validation strategy **(F)**. **(G)** The 5-fold cross-validated ROC curves of the genetic model f52 and the combined models f52s and f52sa predicting the prognosis of STADs. **(H)** The Kaplan–Meier overall survival curves of the TCGA STAD cases stratified by presence or absence of MUC16 mutations. Gehan–Breslow–Wilcoxon tests were performed to compare the overall survivals, and the *p*-values were shown in context.

The overall survival of the included TCGA STAD cases appeared poor, with a median of 805 days (Figure 1A). The cases were classified into good and poor prognostic groups with identical sample size (each with 40 cases) based on a cutoff survival period (573 days), and somatic gene mutation rates were compared between the groups (Supplementary Figure S1A). In total, 52 genes were identified with most striking difference (EBT, *p* < 0.20) (Supplementary Table S3). A random resampling procedure further indicated that these genes were stably associated with STAD prognosis (50/52 with the largest recurrence among the top 50 genes of smallest *p*-values for each resampling test; Supplementary Table S3). Genes involved in collagen chain trimerization were significantly enriched (Supplementary Figure S1B; Fisher’s Exact, FDR = 0.013). Most of the genes (82.7%) were reported to be associated with cancers and 16 (30.8%) with gastric cancer, including MUC16, for which higher mutation rates were recently found to be associated with prognosis and the immune therapy outcome of gastric cancer (Li et al., 2018; Supplementary Table S3). With subsets or all of the 52 genes as features, SVM models were trained to predict the tumor prognosis. Generally, the model performance improved as the number of features increased (Figure 1B). The 52-gene model (f52) could classify the cases into good and poor prognostic groups most accurately, with average 5-fold cross-validated accuracy (*ACC*), area under the receiver operating characteristic (ROC) curves (*AUC*), and Mathews Correlation Coefficient (*MCC*) of 0.81, 0.82, and 0.64, respectively (Supplementary Table S4). Cases classified by the model f52 showed significantly different overall survival (Figure 1C; Gehan–Breslow–Wilcoxon test, *p* = 3e-07).

To test whether the observed mutation–prognosis association was biased by tumor mutation load (TML), we compared the TML distribution between the cases with good and poor prognosis. However, neither total TML nor missense TML showed significant difference between the two groups of cases either classified by the median survival time or predicted by the f52 model (Figure 1D and Supplementary Figure S2; Wilcoxon rank-sum test, *p* > 0.05). Distribution analysis on clinical factors of the training cases demonstrated that clinical TNM stage could be a significant co-founding factor (Supplementary Figure S3). We developed a model featured by stage information, and found that its performance was far inferior to that of f52, despite its ability in classifying the cases into two groups with significantly different overall survival (Supplementary Figure S4, Supplementary Table S4, and Figure 1E). A model combined the 52 genetic features and stage information, f52s, but achieved better performance (Supplementary Table S4), which could classify the cases into two groups with more significant survival difference (Figure 1F; Gehan–Breslow–Wilcoxon test, *p* = 7e-08). The models further integrated with other clinical information-based features (e.g., anatomic site, f52sa), however, performed not better than f52s (Figure 1G and Supplementary Table S4).

MUC16 was recently reported to be associated with the prognosis of gastric cancer (Li et al., 2018). The gene was also included in our multi-gene feature list. We also found that the MUC16 prognosis-prediction model can classify the cases into two prognostic groups, but the significance was much lower than our multi-gene models (Figure 1H; Gehan–Breslow–Wilcoxon test, *p* = 0.03). Other performance measures further demonstrated the superiority of multi-gene models over the individual MUC16 model (Supplementary Figure S5 and Supplementary Table S4).

Taking together, we identified a list of genes, whose somatic mutation profile could be used for effective prediction of prognosis for TCGA STAD cases.

### Somatic Mutation Indicators for Poor Prognosis of TCGA STADs

**FIGURE 2:**
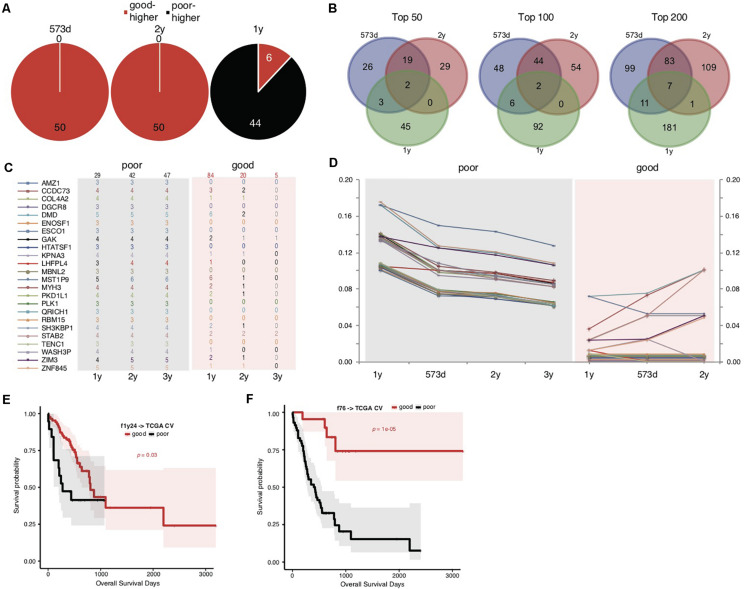
Somatic mutation indicators for poor prognosis of TCGA STADs. **(A)** The distribution of top 50 significant genes of good and poor prognosis groups stratified with different cutoff survival time. Genes with higher mutation rates were counted for either the good or poor prognosis group and represented as “good-higher” or “poor-higher,” respectively. The total number was also indicated. **(B)** The consistent intersect among the top significant genes (50, 100, or 200) identified by 573-day, 1-year, and 2-year stratifications. **(C,D)** The number of cases with mutation **(C)** and the mutation rate changes **(D)** of the top 24 significant genes selected in the good and poor prognostic groups stratified by 1 year along with overall survival time. The total case number for either prognostic group stratified by each survival cutoff was shown on the top of **(C)**. **(E,F)** The Kaplan–Meier overall survival curves of the TCGA STAD cases stratified by f1y24 **(E)** or the combined f76 model **(F)** with a 5-fold cross-validation strategy. Gehan–Breslow–Wilcoxon tests were performed to compare the overall survivals, and the *p*-values were shown in context.

We noticed that the f52 model showed lower classifying power for short-term prognosis of TCGA STAD cases (Figure 1C). All of the 52 genes were also found with higher mutation rates in cases with good prognosis (Supplementary Table S3). Finally, the stratification for STAD prognosis was based on the median overall survival, and it would be also interesting to observe the dynamic changes of gene mutation rates between groups stratified with different cutoff survival times. To this end, we grouped the cases using overall survival of 1, 2, and 3 years as prognosis cutoff respectively besides the median and compared the gene mutation rates. Similar to the comparison results based on 573 days, an absolute majority of top significant genes showed higher mutation rates in the group of good prognosis than that of poor prognosis stratified by 2-year overall survival (Figure 2A; 3-year not shown due to the very limitation of case number for good prognosis). For 1-year stratification, however, the results demonstrated a contrary trend, i.e., most of the top significant genes showing higher mutation rates in the poor-prognosis group (Figure 2A). The consistent intersect between the top significant genes (50, 100, or 200) of 573-day and 2-year stratification was much larger than that between 573-day or 2-year and 1-year (Figure 2B).

To further explore the possible factors causing the observed contrary trends, we identified genes with the most strikingly different mutation rates (with a minimal difference of 10%) between poor and good prognostic groups stratified by 1 year, and observed the mutation rate changes along with overall survival time (Figures 2C,D and Supplementary Table S5). The results suggested that all of these (24) genes inclined to mutate in cases with the poorest prognosis (<1-year overall survival) (Figures 2C,D). As control, the genes showed no or much fewer mutations in cases with good prognosis, and the case number with mutations or mutation rates decreased generally for the patients with longer prognosis (Figures 2C,D).

The above results suggested that these gene mutations could be indicators for poorest prognosis. As validation, we used these genes as features and trained models based on 1-year-stratified TCGA training data (Supplementary Table S4). The 5-fold cross-validated results suggested that the optimized model (f1y24) could distinguish the cases with different prognoses in spite of a weaker distinguishing power (Figure 2E; Gehan–Breslow–Wilcoxon test, *p* = 0.03). Compared to f52, f1y24 did show better performance for the short-term prognosis classification (Figure 2E). Combination of the 24 short-term gene markers and 52 medium- and long-term markers generated a new model, f76, which showed a balanced classification power for both short-term and long-term prognosis classification, although the general significance was not comparable to f52 (Supplementary Table S4 and Figure 2F; Gehan–Breslow–Wilcoxon test, *p* = 1e-05).

### Heterogeneity of Prognosis-Associated Genetic Signatures Between TCGA and Chinese STAD Cohorts

**FIGURE 3:**
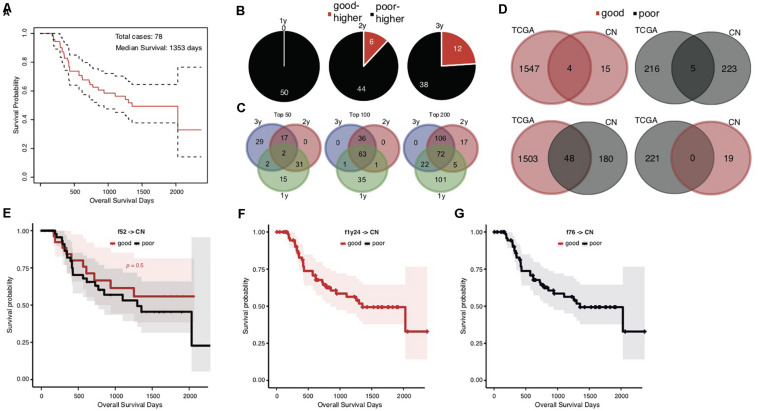
Heterogeneity of prognosis-associated genetic signatures between TCGA and Chinese STAD cohorts. **(A)** The Kaplan–Meier overall survival curve of the Chinese STAD cases, with a median of 1353 days. **(B)** Distribution of the top 50 significant genes with different mutation rates between the good and poor prognostic groups stratified by different cutoff survival times. Genes with higher mutation rates were counted for either the good or poor prognosis group and represented as “good-higher” or “poor-higher,” respectively. The total number was also indicated. **(C)** The consistent intersect among the top significant genes (50, 100, or 200) identified by 1-, 2-, and 3-years stratifications. **(D)** The consistent intersect between the prognosis-associated gene mutation signatures of TCGA and Chinese STAD cohorts. Genes were merged for the 1-year, 2-year, and 573-day stratifications for the TCGA cohort and merged for the 1-year, 2-year, and 3-year stratifications for the Chinese cohort in the first place. **(E–G)** The Kaplan–Meier overall survival curves of the Chinese STAD cases classified with different models built on the TCGA training data.

The overall survival of the Chinese cohort with 78 STAD cases appeared better than the TCGA cohort, with a median of 1353 days (Figure 3A). We also stratified the Chinese cases into good and poor prognostic groups according to 1-, 2-, and 3-years, and median overall survival, respectively. Different from TCGA results, the top significant genes showed large consistence between 1-year and other survival time stratifications (Figures 3B,C).

To our surprise, the Chinese and TCGA cohorts showed an unexpected heterogeneity on the prognosis-associated gene mutation signatures. Very few common genes were identified in both cohorts with either higher or lower mutation rates in good prognostic groups (Figure 3D, upper; 4 with higher and 5 with lower mutation rates in good prognostic groups). More genes even showed the contrary trends in the TCGA and Chinese cohorts, e.g., higher mutation rates in the good prognostic group of TCGA cohort and the poor prognostic group of Chinese cohort (Figure 3D, lower; 48 genes). Further analysis for ethnicity stratification of the TCGA cases was precluded since the number of included Asian cases was too limited, and the secondary prognosis stratification and mutation rate comparison were infeasible.

This dramatic genetic heterogeneity could likely make the TCGA-based prognosis prediction models ineffective in application for the Chinese cohort. The application of the f52, f1y24, and f76 models confirmed the following assumption: none of them could well classify the Chinese cases into groups with different prognosis (Figures 3E–G; Gehan–Breslow–Wilcoxon test for f52, *p* = 0.5; f1y24 and f76 classifying all Chinese cases as good and poor prognosis, respectively).

### Common Signatures Effectively Predict STAD Prognosis of Both TCGA and Chinese Cohorts

**FIGURE 4:**
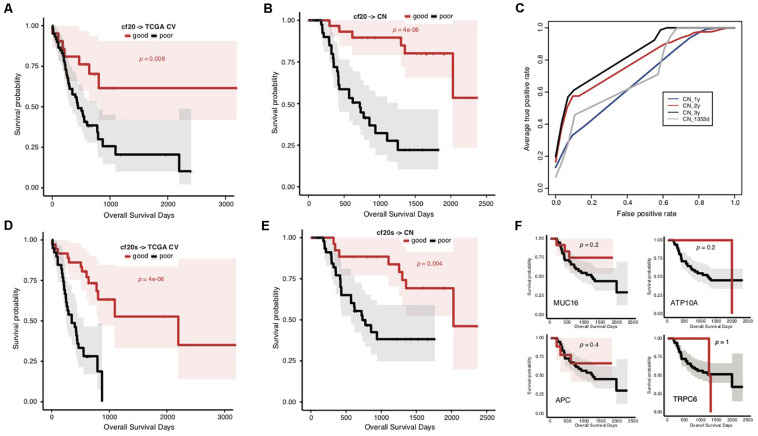
Prediction of STAD prognosis with the genetic models based on 20 common somatic mutation features. **(A)** The Kaplan–Meier overall survival curves of the TCGA STAD cases classified by cf20 with a 5-fold cross-validation strategy. **(B)** The Kaplan–Meier overall survival curves of the Chinese STAD cases stratified by the cf20 model. **(C)** The 5-fold cross-validated ROC curves of cf20 model predicting the prognosis of Chinese STAD cohorts stratified by different cutoff survival time. **(D)** The Kaplan–Meier overall survival curves of the TCGA STAD cases classified by the cf20s model which was combined 20 common-gene features and TNM stage information. **(E)** The Kaplan–Meier overall survival curves of the Chinese STAD cases stratified by the combined cf20s model. **(F)** The Kaplan–Meier overall survival curves of the Chinese STAD cases stratified by models based on example individual genes, including MUC16, ATP10A, APC, and TRPC6.

To overcome the generalization drawbacks of the prognosis prediction models based on individual cohorts due to the genetic heterogeneity, we came up with a new strategy to identify and test a list of new signatures by screening the genes with the same change trend of somatic mutation rates between prognostic groups in the TCGA and Chinese cohorts. Genes were extracted with different levels of mutation rate difference (≥15%, ≥10%, and ≥5%) between prognostic groups for both cohorts stratified, respectively, and the common ones were further identified correspondingly to serve as signatures. To reduce the biases caused by imbalanced sample size between groups, the prognostic groups were stratified by an overall survival period of 576 days for the TCGA cohort and 3 years for the Chinese cohort, respectively, with which the two groups in either cohort showed the identical sample size. There were 0, 4 (*MUC16*, *ATP10A*, *MPDZ*, and *VPS13A*) and 20 genes showing ≥15%, ≥10%, and ≥5% mutation rate differences between prognostic groups for both cohorts with the same direction (Supplementary Table S6). Furthermore, the 20 genes (with ≥ 5% mutation rate difference) were tested for the prognosis prediction performance as signatures. With these common feature genes, we trained a prognosis prediction model (cf20) based on the TCGA training data stratified with the median survival time. The 5-fold cross-validation performance on TCGA data was not comparable to f52, however, it remained to be effective in classifying the data into two prognostic groups (Figure 4A; Gehan–Breslow–Wilcoxon test, *p* = 0.008). The model appeared much more effective in prognosis prediction of the Chinese cohort (Figure 4B; Gehan–Breslow–Wilcoxon test, *p* = 4e-06). It consistently showed good performance to predict the different stratifications of prognosis for the Chinese cohort, especially for 3- and 2-year prognosis (Figure 4C).

We also integrated the TNM staging information in cf20 to generate a new model, cf20s. For TCGA data and based on the 5-fold cross-validation evaluation, cf20s apparently outperformed cf20 (Figure 4D; Gehan–Breslow–Wilcoxon test for cf20s, *p* = 4e-06). When testing in the Chinese cohort, however, the performance deteriorated, in spite that it remained effective to predict the prognosis (Figure 4E; Gehan–Breslow–Wilcoxon test, *p* = 4e-06). Both cf20 and cf20s, however, outperformed the single-gene models in predicting the prognosis of the STAD cases (Figure 4F).

## Discussion

In this research, we found the association between overall prognosis of STADs and somatic gene mutations from the TCGA cohort. Despite that the rate comparison-based feature extraction strategy involved division of the cases into different prognostic groups according to a survival cutoff preset subjectively, the model (f52, median survival as cutoff) could well classify the cases into two groups with significantly differentiated survival (Figures 1B,C). It is noteworthy that 5-fold cross validation was used for assessment of model performance, independent between each training subset and testing subset, and the survival comparison was performed between the predicted groups for all testing cases. Therefore, the observed association was not biased by the model-training scheme. Except tumor stage, other possible co-founding clinical factors, including sex, anatomic site, and histopathology, did not show a biased distribution between the prognostic groups. The TNM staging system could predict STAD prognosis independently but was not comparable to the f52 genetic model (Figure 1E and Supplementary Figure S4). Combination of the genetic features and stage information improved the prognosis-classifying performance significantly (Figure 1F). Therefore, as for the TCGA cohort, the prognosis is associated with genetic factors.

It was noted that all the 52 genes with most significant difference showed higher mutation rates in the good prognosis group of TCGA cases stratified by the median survival time (Figure 2A). It was consistent with previous findings in lung adenocarcinomas (Yu et al., 2019). Recently, a study identified the association between higher MUC16 gene mutation rate and better prognosis of STADs. Meanwhile, the more frequent MUC16 mutation was associated with a higher TML (Li et al., 2018). Maruvka et al. argued that a larger MUC16 mutation frequency could only be an accompanying result of high TML (Maruvka et al., 2019). MUC16 was also present in our 52-gene list. We suspected that the list of signature genes with different mutation rates in prognostic groups could be merely caused by different TMLs. However, no significant difference was detected for either TMLs or missense TMLs between the prognosis groups of the TCGA data (Figure 1D and Supplementary Figure S2). Interestingly, we noticed that, for TML or missense TML, although there was no difference in the medians or lower quartile, the good prognosis group always showed a larger upper quartile (Figure 1D). Therefore, a higher TML could be an important but not unique factor predicting better prognosis. The identified signatures could partially represent TML difference and also represent other unknown mechanisms influencing the prognosis of STAD.

With the analytic strategy in this study, we also got an interesting finding that the composition of genes and the direction of mutation rate difference between groups stratified by 1-year survival were totally different from those identified for median (573-day) or 2-year stratification (Figures 2A,B). The latter two stratifications showed larger consistency between each other (Figures 2A,B). A list of genes was identified with different mutation rates between prognostic groups stratified by 1 year, which showed more frequent mutations in the group of poor prognosis (Figure 2C). These genes tend to mutate in cases of poorest prognosis (Figures 2C,D), unlike those identified in median (or 2-year) stratification for which the mutation rate showed a linear correlation with overall survival period generally. The model trained with the 1-year gene features could only distinguish the cases with poorest prognosis (Figure 2E), further demonstrating that the mutations of these signatures could be the indicators of very poor prognosis of STAD.

Heterogeneity of STADs and their genetics was not surprisingly identified between cohorts, and yet the dramatic difference of prognosis-associated genetic signatures between the TCGA and Chinese cohort was unexpected (Figure 3D). Direct application of the signatures and models trained in the TCGA cohort showed an awful performance in prognosis prediction of the Chinese cohort (Figures 3E–G). There was a large heterogeneity of genes with mutation rate difference identified from the two cohorts. Many genes even showed a contrary trend for the mutation rate in prognostic groups (Figure 3D). We attempted to isolate the Asian cases from the TCGA cohort but failed to evaluate the gene mutation rates within different prognosis groups due to the very limited number of the cases. It remains to be clarified whether the heterogeneity between cohorts is related with ethnicity of STAD cases. Two prognostic biomarker genes, BRCA2 and MUC16 (Chen et al., 2015; Li et al., 2018), were found with a mutation rate difference between the good and poor prognostic groups, and with the same trend in the two cohorts. We modified the signature-identification strategy, with an attempt to find out all the common genes with a consistent mutation difference between prognostic groups within each cohort. In total, 20 genes were identified, including MUC16 and BRCA2. A model (cf20) was trained with these genes as features and the TCGA cohort as training data. The model well predicted the prognosis of both TCGA cases based on a cross-validation evaluation, and the Chinese cases independently (Figures 4A–C). The multi-gene model also outperformed the ones based on individual genes strikingly (Figure 4F). However, the problems of over-fitting cannot be totally excluded despite of the use of only TCGA data for model training and cross-validation evaluation, and the Chinese cohort as an independent validation dataset, because the signatures were identified using both the cohorts. The effective sample sizes for the cohorts (especially the Chinese cohort) were too small so that they were hardly further divided, and therefore resampling or cross-validation-based feature identification strategies appeared difficult. It would be better to, but currently we cannot, find one or more independent STAD datasets (with both gene mutation profiling data and clinical follow-up information) to make further assessment. New larger datasets are also in need to further evaluate the potential heterogeneity caused by human ethnicity and develop more ethnicity-specific models like the f52 for the TCGA population.

Besides somatic mutation signatures, germline variants could also be associated with tumor prognosis. Recently, Milanese et al. reported different germline variants in recurred and non-recurred patients of breast cancers (Milanese et al., 2019). These signature germline variants could potentially facilitate the formation of the pro-tumorigenic environment by impairing adaptive and innate immune pathways and could be used for prediction of breast cancer outcomes (Milanese et al., 2019). In another study, Xu et al. (2019) observed negative associations between the number of germline defective genes in natural killer cells and survival time in a variety of cancer types. It is interesting to understand whether there is also heterogeneity between different cohorts for the associations between germline mutations and STAD prognosis. Combination of both germline variants and somatic mutations as well as other signatures, e.g., hypermethylation signatures, and RNA markers, could also further improve the model prediction performance on STAD prognosis.

## Data Availability Statement

The datasets presented in this study can be found in online repositories. The names of the repository/repositories and accession number(s) can be found in the article/Supplementary Material.

## Author Contributions

YW, XC, and DL conceived the project. XL, XH, and YW coordinated the project. XH, HK, QF, and AC collected the data. XL, XH, HK, YH, and YW performed the analysis. XH, HK, and YW developed the models. XL, XH, HK, and YW wrote the first draft. XL, XH, HK, DL, XC, and YW revised the manuscript. All authors approved the final version of manuscript.

## Conflict of Interest

The authors declare that the research was conducted in the absence of any commercial or financial relationships that could be construed as a potential conflict of interest.

## Footnotes

1 https://cran.r-project.org/

## Supplementary Material

FIGURE S1 The general prognosis of TCGA STAD cases and the functional enrichment analysis of the prognosis-associated genes. **(A)** The good and poor prognostic groups of STAD cases within different survival periods, including 1, 2, and 3 years and the median of 573 days. **(B)** Gene Ontology (GO) enrichment analysis of the top 52 genes with significant mutation rate difference between the prognostic groups stratified by the median survival time.

FIGURE S2 TML distribution for different prognostic groups of the TCGA cases. The distribution of TMLs **(A)** and missense TMLs **(B,C)** for TCGA cases of good and poor prognosis groups for the raw TCGA training dataset stratified by 573-day survival or classified by the f52 model. The *p*-value of Wilcoxon rank-sum test was indicated.

FIGURE S3 Distribution analysis on clinical factors of the training cases. Sex **(A)**, anatomic regions of stomach **(B)**, clinical TNM stage **(C)**, and the two main histological types of gastric carcinoma **(D)** were involved. Chi-square tests were performed and the *p*-values were indicated.

FIGURE S4 Performance comparison of the prognosis prediction models based on 52 somatic mutation features and clinical TNM stage information. Specificity (Sp), Sensitivity (Sn), Accuracy (ACC), and Mathews Correlation Coefficient (MCC) were utilized to assess the predictive performance. The model f52 was based on the 5-fold cross-validation results. Pairwise one-tail Student’s *t*-tests were performed, and the *p*-values were indicated.

FIGURE S5 Performance comparison of the prognosis prediction models based on 52 somatic mutation features and MUC16. Specificity (Sp), Sensitivity (Sn), Accuracy (ACC), and Mathews Correlation Coefficient (MCC) were utilized to assess the predictive performance. The model f52 was based on the 5-fold cross-validation results. Pairwise one-tail Student’s *t*-tests were performed, and the *p*-values were indicated.

## References

[B1] Banks M. Graham D. Jansen M. Gotoda T. Coda S. di Pietro M. (2019). British Society of Gastroenterology guidelines on the diagnosis and management of patients at risk of gastric adenocarcinoma. *Gut* 68 1545–1575. 10.1136/gutjnl-2018-318126 31278206 PMC6709778

[B2] Bosman F. T. Carneiro F. Hruban R. H. Theise N. D. (2010). *WHO Classification of Tumours of the Digestive System*, 4th Edn Geneva: World Health Organization.

[B3] Boussioutas A. Li H. Liu J. Waring P. Lade S. Holloway A. J. (2003). Distinctive patterns of gene expression in premalignant gastric mucosa and gastric cancer. *Cancer Res.* 63 2569–2577. 12750281

[B4] Bray F. Ferlay J. Soerjomataram I. Siegel R. L. Torre L. A. Jemal A. (2018). Global cancer statistics 2018: GLOBOCAN estimates of incidence and mortality worldwide for 36 cancers in 185 co*f*untries. *CA Cancer J Clin.* 68 394–424. 10.3322/caac.21492 30207593

[B5] Cancer Genome, Atlas Research, and Network. (2014). Comprehensive molecular characterization of gastric adenocarcinoma. *Nature* 513 202–209. 10.1038/nature13480 25079317 PMC4170219

[B6] Charalampakis N. Economopoulou P. Kotsantis I. Tolia M. Schizas D. Liakakos T. (2018). Medical management of gastric cancer: a 2017 update. *Cancer Med.* 7 123–133. 10.1002/cam4.1274 29239137 PMC5773977

[B7] Chen K. Yang D. Li X. Sun B. Song F. Cao W. (2015). Mutational landscape of gastric adenocarcinoma in Chinese: implications for prognosis and therapy. *Proc. Natl. Acad. Sci. U.S.A.* 112 1107–1112. 10.1073/pnas.1422640112 25583476 PMC4313862

[B8] Cho H. J. Lee S. Ji Y. G. Lee D. H. (2018). Association of specific gene mutations derived from machine learning with survival in lung adenocarcinoma. *PLoS One* 13:e0207204. 10.1371/journal.pone.0207204 30419062 PMC6231670

[B9] Choi E. Roland J. T. Barlow B. J. O’Neal R. Rich A. E. Nam K. T. (2014). Cell lineage distribution atlas of the human stomach reveals heterogeneous gland populations in the gastric antrum. *Gut* 63 1711–1720. 10.1136/gutjnl-2013-305964 24488499 PMC4117823

[B10] Cristescu R. Lee J. Nebozhyn M. Kim K. M. Ting J. C. Wong S. S. (2015). Molecular analysis of gastric cancer identifies subtypes associated with distinct clinical outcomes. *Nat. Med.* 21 449–456. 25894828 10.1038/nm.3850

[B11] Hui X. Hu Y. Sun M. A. Shu X. Han R. Ge Q. (2017). EBT: a statistic test identifying moderate size of significant features with balanced power and precision for genome-wide rate comparisons. *Bioinformatics* 33 2631–2641. 10.1093/bioinformatics/btx294 28472273

[B12] Laurén P. (1965). The two histological main types of gastric carcinoma: diffuse and so-called intestinal-type carcinoma. *Acta Pathol. Microbiol. Scand.* 64 31–49. 10.1111/apm.1965.64.1.31 14320675

[B13] Lee D. W. Han S. W. Cha Y. Bae J. M. Kim H. P. Lyu J. (2017). Association between mutations of critical pathway genes and survival outcomes according to the tumor location in colorectal cancer. *Cancer* 123 3513–3523. 10.1002/cncr.30760 28513830

[B14] Lei Z. Tan I. B. Das K. Deng N. Zouridis H. Pattison S. (2013). Identification of molecular subtypes of gastric cancer with different responses to PI3-kinase inhibitors and 5-fluorouracil. *Gastroenterology* 145 554–565. 10.1053/j.gastro.2013.05.010 23684942

[B15] Li X. Pasche B. Zhang W. Chen K. (2018). Association of MUC16 mutation with tumor mutation load and outcomes in patients with gastric Cancer. *JAMA Oncol.* 4 1691–1698. 30098163 10.1001/jamaoncol.2018.2805 PMC6440715

[B16] Loi S. Michiels S. Lambrechts D. Fumagalli D. Claes B. Kellokumpu-Lehtinen P. L. (2013). Somatic mutation profiling and associations with prognosis and trastuzumab benefit in early breast cancer. *J. Natl. Cancer Inst.* 105 960–967. 10.1093/jnci/djt121 23739063 PMC3699437

[B17] Maruvka Y. E. Haradhvala N. J. Getz G. (2019). Analyzing frequently mutated genes and the association with tumor mutation load. *JAMA Oncol.* 5:577. 10.1001/jamaoncol.2019.0127 30844029

[B18] Milanese J. S. Tibiche C. Zou J. Meng Z. Nantel A. Drouin S. (2019). Germline variants associated with leukocyte genes predict tumor recurrence in breast cancer patients. *NPJ Precis Oncol.* 3:28. 10.1038/s41698-019-0100-7 PMC6825127 31701019

[B19] Ni X. Tan Z. Ding C. Zhang C. Song L. Yang S. (2019). A region-resolved mucosa proteome of the human stomach. *Nat. Commun.* 10:39. 10.1038/s41467-018-07960-x PMC6318339 30604760

[B20] Oh S. C. Sohn B. H. Cheong J. H. Kim S. B. Lee J. E. Park K. C. (2018). Clinical and genomic landscape of gastric Cancer with a mesenchymal phenotype. *Nat. Commun.* 9:1777. 10.1038/s41467-018-04179-8 PMC5934392 29725014

[B21] Ooi W. F. Xing M. Xu C. Yao X. Ramlee M. K. Lim M. C. (2016). Epigenomic profiling of primary gastric adenocarcinoma reveals super-enhancer heterogeneity. *Nat. Commun.* 7:12983. 10.1038/ncomms12983 PMC5052795 27677335

[B22] Shen L. Shan Y. S. Hu H. M. Price T. J. Sirohi B. Yeh K. H. (2013). Management of gastric cancer in asia: resource-stratified guidelines. *Lancet Oncol.* 14:e535-47. 10.1016/S1470-2045(13)70436-4 24176572

[B23] Soybel D. I. (2005). Anatomy and physiology of the stomach. *Surg. Clin. North Am.* 85 875–894. 10.1016/j.suc.2005.05.009 16139026

[B24] Tan I. B. Ivanova T. Lim K. H. Ong C. W. Deng N. Lee J. (2011). Intrinsic subtypes of gastric cancer, based on gene expression pattern, predict survival and respond differently to chemotherapy. *Gastroenterology* 141 476–485. 21684283 10.1053/j.gastro.2011.04.042 PMC3152688

[B25] Tran P. N. Sarkissian S. Chao J. Klempner S. J. (2017). PD-1 and PD-L1 as emerging therapeutic targets in gastric cancer: current evidence. *Gastrointest. Cancer* 7 1–11. 10.2147/gictt.s113525 28757801 PMC5533281

[B26] Waldum H. L. Fossmark R. (2018). Types of gastric carcinomas. *Int. J. Mol. Sci.* 19:4109. 10.3390/ijms19124109 30567376 PMC6321162

[B27] Xu X. Li J. Zou J. Feng X. Zhang C. Zheng R. (2019). Association of germline variants in natural killer cells with tumor immune microenvironment subtypes, tumor-infiltrating lymphocytes, immunotherapy response, clinical outcomes, and Cancer risk. *JAMA Netw. Open* 2:e199292. 10.1001/jamanetworkopen.2019.9292 31483464 PMC6727785

[B28] Yu J. Hu Y. Xu Y. Wang J. Kuang J. Zhang W. (2019). LUADpp: an effective prediction model on prognosis of lung adenocarcinomas based on somatic mutational features. *BMC Cancer* 19:263. 10.1186/s12885-019-5433-7 30902072 PMC6431052

[B29] Zhang P. Yang M. Zhang Y. Xiao S. Lai X. Tan A. (2019). Dissecting the Single-Cell transcriptome network underlying gastric premalignant lesions and early gastric Cancer. *Cell Rep.* 27 1934–1947. 31067475 10.1016/j.celrep.2019.04.052

[B30] Zhang S. Xu Y. Hui X. Yang F. Hu Y. Shao J. (2017). Improvement in prediction of prostate cancer prognosis with somatic mutational signatures. *J. Cancer* 8 3261–3267. 10.7150/jca.21261 29158798 PMC5665042

