# Supplementary figures and images for: A Multi-Gene Model Effectively Predicts the Overall Prognosis of Stomach Adenocarcinomas With Large Genetic Heterogeneity Using Somatic Mutation Features
Source: Front Genet. 2020 Aug 26;11:940. doi: 10.3389/fgene.2020.00940 (PMC7479248; doi:10.3389/fgene.2020.00940)

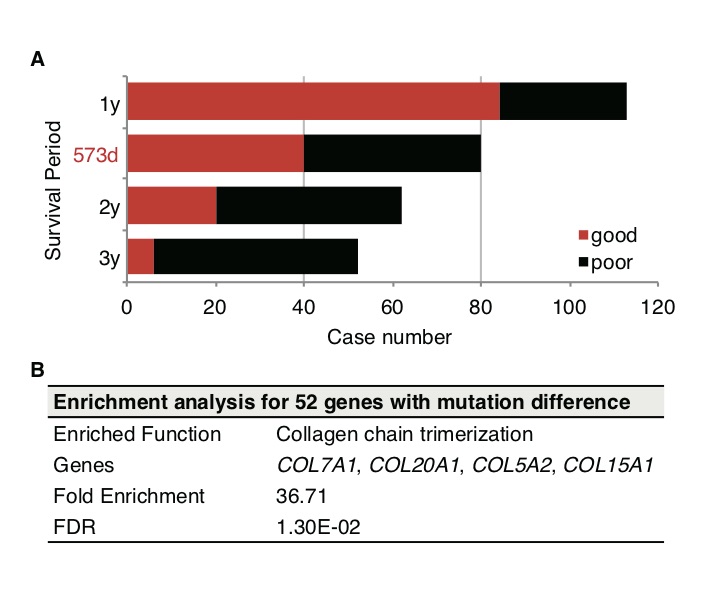

Supplement: FIGURE S1 — The general prognosis of TCGA STAD cases and the functional enrichment analysis of the prognosis-associated genes. (A) The good and poor prognostic groups of STAD cases within different survival periods, including 1, 2, and 3 years and the median of 573 days. (B) Gene Ontology (GO) enrichment analysis of the top 52 genes with significant mutation rate difference between the prognostic groups stratified by the median survival time. [file Image_1.JPEG]

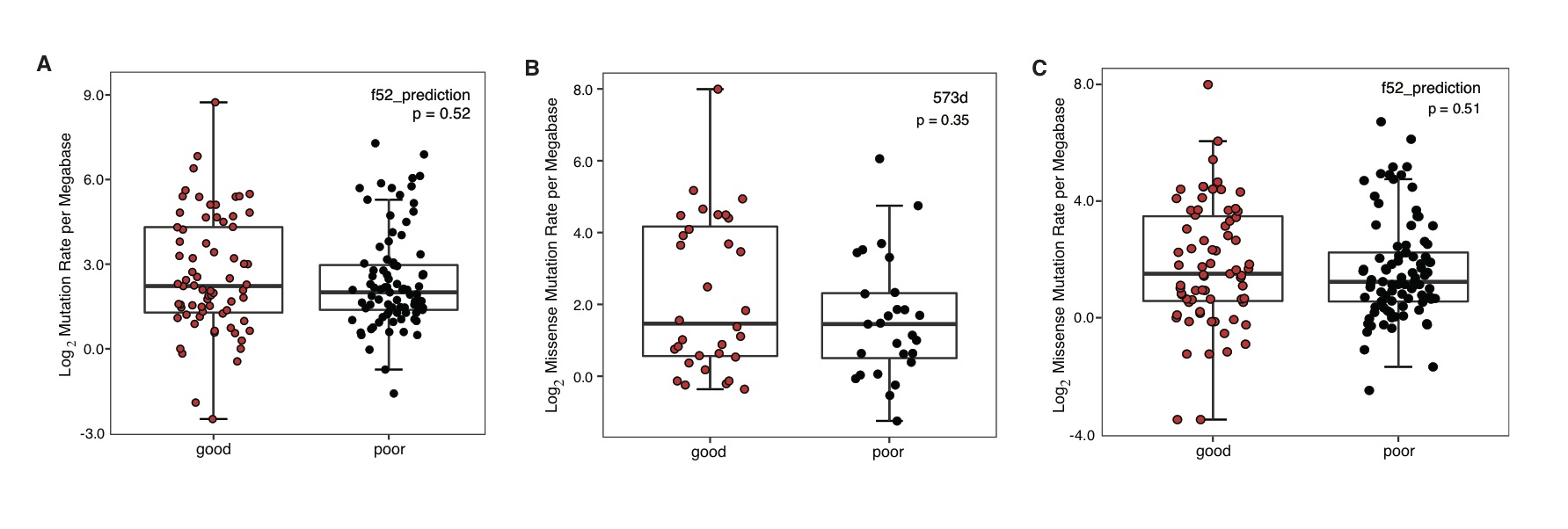

Supplement: FIGURE S2 — TML distribution for different prognostic groups of the TCGA cases. The distribution of TMLs (A) and missense TMLs (B,C) for TCGA cases of good and poor prognosis groups for the raw TCGA training dataset stratified by 573-day survival or classified by the f52 model. The p-value of Wilcoxon rank-sum test was indicated. [file Image_2.JPEG]

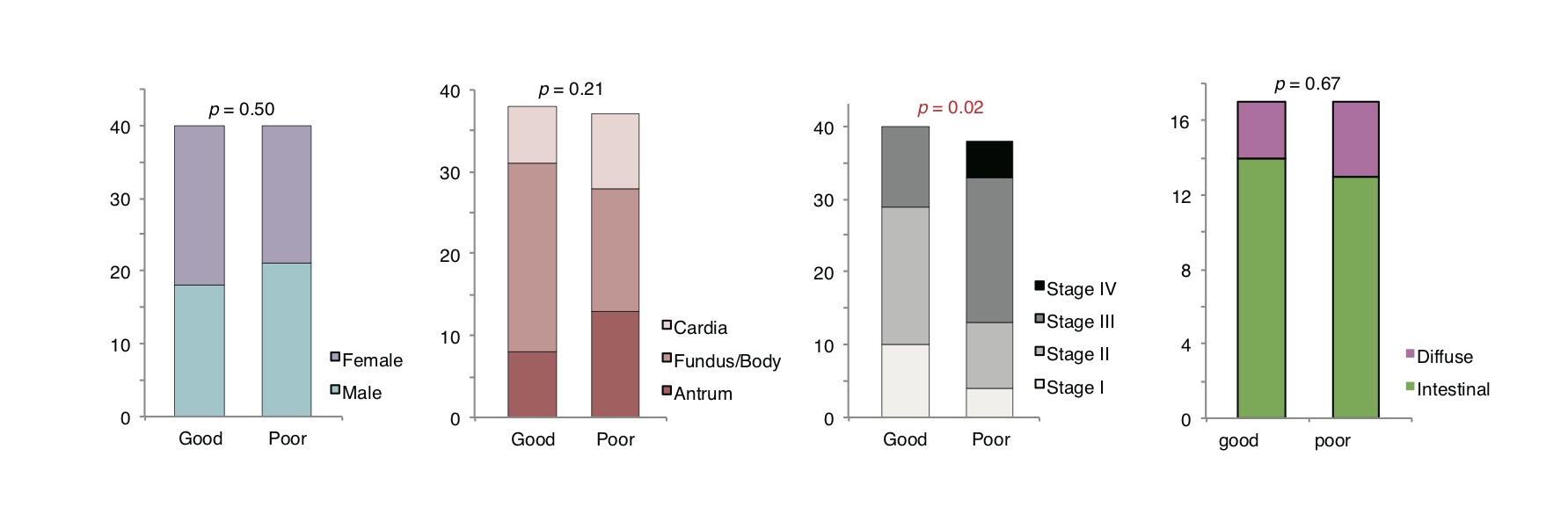

Supplement: FIGURE S3 — Distribution analysis on clinical factors of the training cases. Sex (A), anatomic regions of stomach (B), clinical TNM stage (C), and the two main histological types of gastric carcinoma (D) were involved. Chi-square tests were performed and the p-values were indicated. [file Image_3.JPEG]

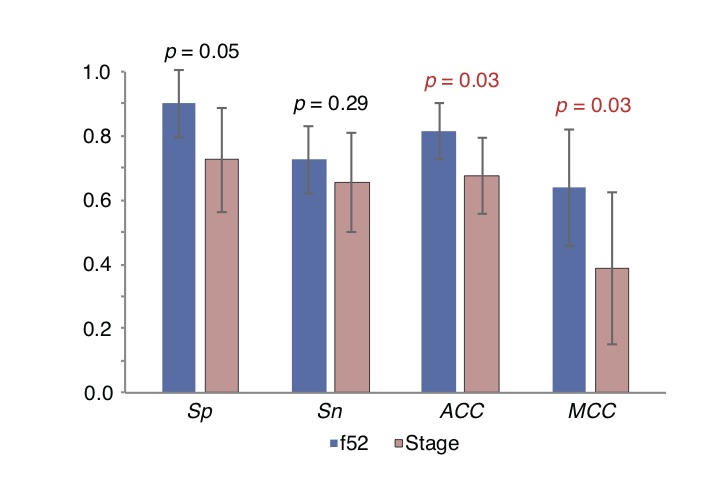

Supplement: FIGURE S4 — Performance comparison of the prognosis prediction models based on 52 somatic mutation features and clinical TNM stage information. Specificity (Sp), Sensitivity (Sn), Accuracy (ACC), and Mathews Correlation Coefficient (MCC) were utilized to assess the predictive performance. The model f52 was based on the 5-fold cross-validation results. Pairwise one-tail Student’s t-tests were performed, and the p-values were indicated. [file Image_4.JPEG]

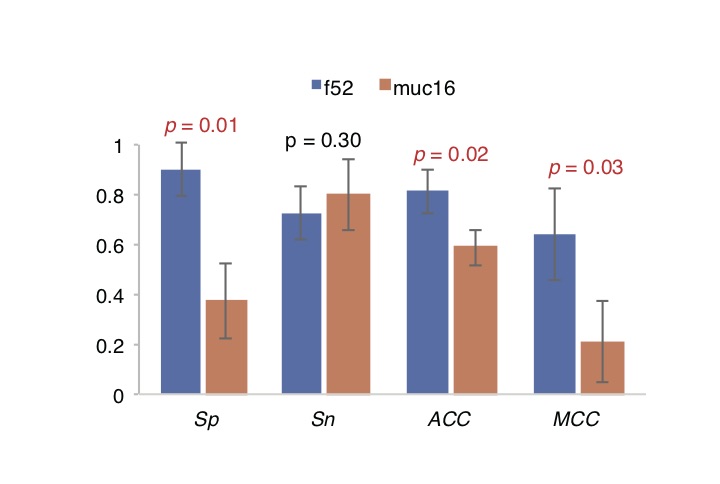

Supplement: FIGURE S5 — Performance comparison of the prognosis prediction models based on 52 somatic mutation features and MUC16. Specificity (Sp), Sensitivity (Sn), Accuracy (ACC), and Mathews Correlation Coefficient (MCC) were utilized to assess the predictive performance. The model f52 was based on the 5-fold cross-validation results. Pairwise one-tail Student’s t-tests were performed, and the p-values were indicated. [file Image_5.JPEG]
