# Supplementary material for: A Multi-Gene Model Effectively Predicts the Overall Prognosis of Stomach Adenocarcinomas With Large Genetic Heterogeneity Using Somatic Mutation Features
Source: Front Genet. 2020 Aug 26;11:940. doi: 10.3389/fgene.2020.00940 (PMC7479248; doi:10.3389/fgene.2020.00940)
Supplement: Supplementary file 6 [file Table_1.DOCX]

**Table S1. Summary of the TCGA STAD cases used for this study**

| **Clinic Features** |  | **Cases** |
| --- | --- | --- |
| **Sex** | Male | 81 |
|  | Female | 61 |
| **Race** | White | 111 |
|  | Asian | 15 |
|  | Others/ Not Available | 16 |
| **Subdivision** | Gastroesophageal Junction | 7 |
|  | Cardia/Proximal | 18 |
|  | Fundus/Body | 67 |
|  | Antrum/Distal | 44 |
|  | Not Available | 6 |
| **Stage** | IB | 7 |
|  | IA | 20 |
|  | II | 52 |
|  | III | 55 |
|  | IV | 6 |
|  | Not Available | 2 |
| **Total** |  | 142 |
