# Supplementary material for: A Multi-Gene Model Effectively Predicts the Overall Prognosis of Stomach Adenocarcinomas With Large Genetic Heterogeneity Using Somatic Mutation Features
Source: Front Genet. 2020 Aug 26;11:940. doi: 10.3389/fgene.2020.00940 (PMC7479248; doi:10.3389/fgene.2020.00940)
Supplement: Supplementary file 7 [file Table_2.DOCX]

**Table 2. Signature genes with different mutation rates between the strata of clinical features**

| **Clinic Features** | | **Top Genes With Higher Somatic Mutation Rates** |
| --- | --- | --- |
| **General** |  | *TTN*, *PCDHAC2*, *PCDHGC5*, *TP53*, *MUC16*, *SYNE1*, *CSMD3* |
| **Subdivision** | Cardia | *INO80D* (🡹), *RYR2* (🡻) |
|  | Fundus/Body | *PRR12* (🡹) |
|  | Antrum | *EHMT1* (🡹), *GPR98* (🡻) |
| **Sex** | Female | *PRKDC*, *SRCAP*, *PARD3B*, *PTCH2*, *RBM27*, *UBC* (🡹) |
