# Supplementary material for: A Multi-Gene Model Effectively Predicts the Overall Prognosis of Stomach Adenocarcinomas With Large Genetic Heterogeneity Using Somatic Mutation Features
Source: Front Genet. 2020 Aug 26;11:940. doi: 10.3389/fgene.2020.00940 (PMC7479248; doi:10.3389/fgene.2020.00940)
Supplement: Supplementary file 10 [file Table_5.DOCX]

**Genes inclined to mutate in TCGA cases with the poorest prognosis**

AMZ1

CCDC73

COL4A2

DGCR8

DMD

ENOSF1

ESCO1

GAK

HTATSF1

KPNA3

LHFPL4

MBNL2

MST1P9

MYH3

PKD1L1

PLK1

QRICH1

RBM15

SH3KBP1

STAB2

TENC1

WASH3P

ZIM3

ZNF845
