# Supplementary material for: A Multi-Gene Model Effectively Predicts the Overall Prognosis of Stomach Adenocarcinomas With Large Genetic Heterogeneity Using Somatic Mutation Features
Source: Front Genet. 2020 Aug 26;11:940. doi: 10.3389/fgene.2020.00940 (PMC7479248; doi:10.3389/fgene.2020.00940)
Supplement: Supplementary file 11 [file Table_6.DOCX]

**Common genes with similar between-prognosis mutation rate changing direction and significance in different STAD cohorts**

| **Gene** | **RateDiff_CN-3y** | **RateDiff_TCGA-573d** |
| --- | --- | --- |
| MBNL2 | 0.074074074 | 0.075 |
| SPAG9 | 0.111111111 | 0.05 |
| SMAD4 | 0.111111111 | 0.05 |
| MUC16 | -0.148148148 | -0.225 |
| ATP10A | -0.111111111 | -0.1 |
| C14orf118 | 0.074074074 | 0.05 |
| MYBPC3 | 0.074074074 | 0.05 |
| PTPN14 | 0.074074074 | 0.05 |
| TRPC6 | -0.074074074 | -0.1 |
| PKD1L1 | 0.074074074 | 0.05 |
| TDRKH | 0.111111111 | 0.05 |
| APC | -0.074074074 | -0.1 |
| GPR133 | -0.074074074 | -0.1 |
| MPDZ | -0.111111111 | -0.1 |
| VPS13A | -0.111111111 | -0.1 |
| PTPRT | -0.074074074 | -0.125 |
| ADAM22 | -0.074074074 | -0.1 |
| KDM5B | -0.074074074 | -0.1 |
| KIAA0100 | -0.074074074 | -0.1 |
| BRCA2 | -0.052 | -0.125 |

Note: ‘RateDiff’ means the difference of somatic mutation rates between good prognosis group and poor group (good - poor).
